# Microbiome-Driven Risk Stratification and Biotherapeutics in Radiation Enteritis

**DOI:** 10.34133/csbj.0207

**Published:** 2026-08-24

**Authors:** Ziyi Wang, Yihan Chai, Tianyu Lin, Qingyun Zhang, Xuqiu Shen, Yuqing Shi, Fanchao Xu, Yiyin Zhang, Qijiang Mao, Yuelong Liang

**Affiliations:** ^1^ Department of General Surgery, Sir Run Run Shaw Hospital, School of Medicine, Zhejiang University, Hangzhou 310016, China.; ^2^ Department of Computer Science and Technology, School of Information Science and Technology, Beijing Foreign Studies University, Beijing, China.; ^3^ Beijing Lixing Shiyuan Education and Technology Co. Ltd., Beijing, China.; ^4^ Department of Nursing, Sir Run Run Shaw Hospital, Zhejiang University School of Medicine, Hangzhou, China.; ^5^ Department of Breast Center, Peking University People’s Hospital, Peking University, Beijing, China.

## Abstract

Radiation enteritis (RE) is a severe, dose-limiting complication of cancer radiotherapy that affects the therapeutic outcomes of patients and their quality of life. Current risk assessments primarily rely on physical dosimetry. However, this approach inherently ignores the substantial individual variability in radiation susceptibility, creating an urgent clinical need for personalized predictive tools. Emerging evidence suggests that the gut microbiota plays a central role in RE pathogenesis. Ionizing radiation can cause rapid and profound changes in intestinal ecological structure by reducing the number of beneficial microbes while increasing that of harmful pathogens; these factors interact to exacerbate damage to the mucosal barrier and the ongoing inflammatory response. This review synthesizes the multifaceted role the gut microbiome plays in RE, uniquely emphasizing its clinical translational value. Baseline microbial signatures and dynamic multi-omics data, particularly specific taxa and metabolites of the gut microbiota, emerged as powerful predictive biomarkers. These biological signals could inform risk stratification; however, large-scale clinical validation is required. Moreover, integrating microbiome data into machine learning algorithms represents an exploratory approach for toxicity prediction. Crucially, the evaluation of microbiota-targeted therapies reveals an expanding landscape of preclinical and early-phase clinical research. Future interventions may expand beyond conventional probiotics and fecal microbiota transplantation to include experimental therapies such as rationally designed consortia and engineered biotherapeutics. Finally, a strategic roadmap is proposed, utilizing large-scale longitudinal cohorts, high-resolution spatial transcriptomics, and explainable artificial intelligence. These advances may support the transition toward proactive microbiome-guided management strategies.

## Introduction

A variety of therapeutic strategies have been developed in cancer treatment, among which radiation therapy remains one of the most widely used [1]. Intestinal injury following abdominal and pelvic radiotherapy remains common and adversely affects the quality of life and treatment tolerance of patients [2,3]. Radiation enteritis (RE) is a common dose-limiting complication of abdominal and pelvic radiotherapy, categorized into acute (e.g., diarrhea and abdominal pain) and chronic (e.g., fibrosis and strictures) types [3–6] . Given its adverse impacts on patient quality of life and treatment tolerance, developing strategies to mitigate RE while maintaining therapeutic efficacy remains a critical clinical priority. Therefore, developing strategies that can reduce RE while maintaining or enhancing the efficacy of radiation therapy is urgently needed.

**Fig. 1.**
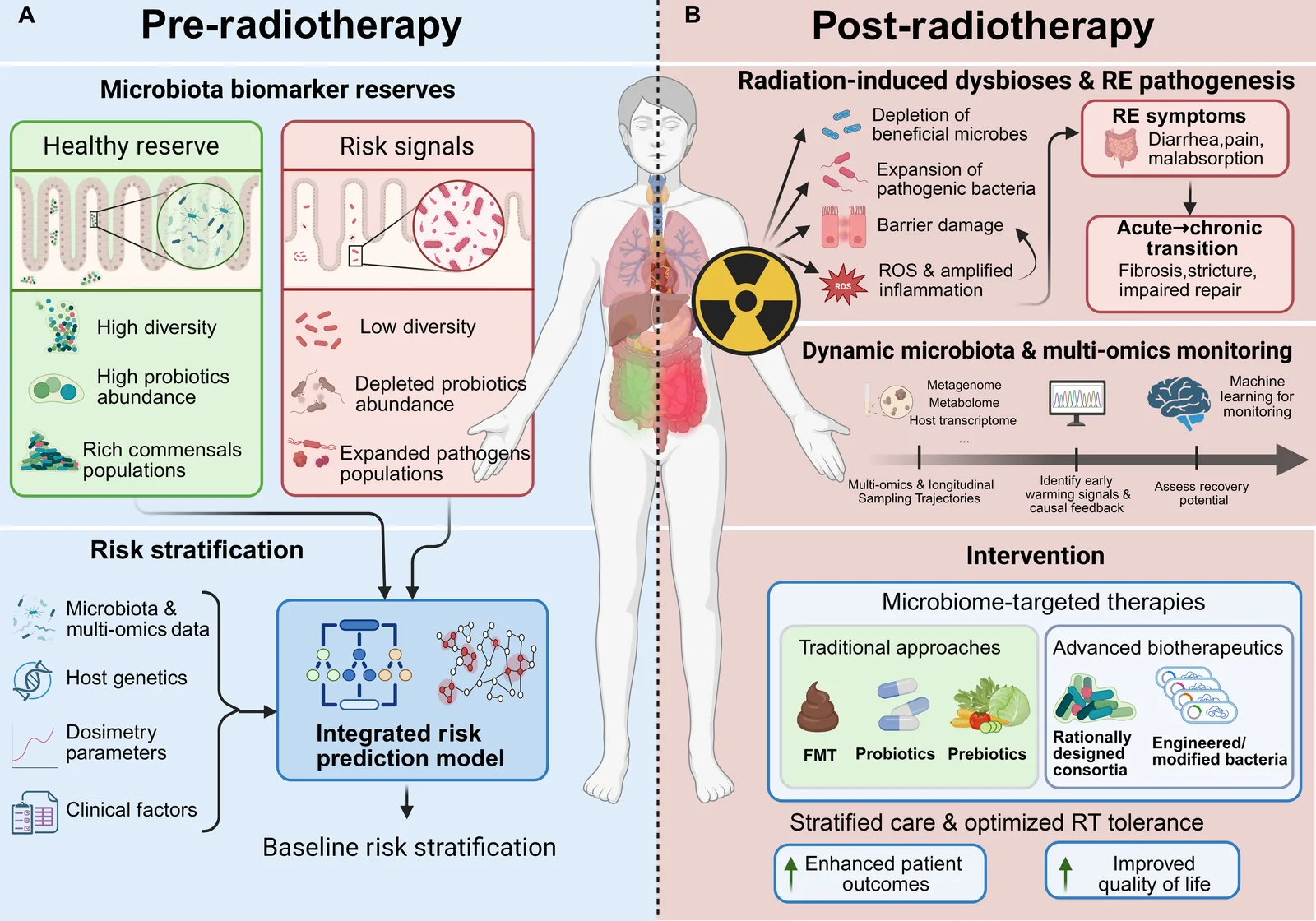
A microbiome-centric framework for RE: From predictive biomarkers and dynamic monitoring to stratified interventions. (A) Pre-radiotherapy: Baseline gut microbiota analysis is combined with clinical and dose data to build a hierarchical risk prediction model based on machine learning methods for early-stage patients. (B) Post-radiotherapy: Radiation-induced dysbiosis acts as a pathological driver of RE. Continuous dynamic monitoring via multi-omics identifies early warning signals, guiding personalized microbiome-targeted interventions to mitigate toxicity and enhance clinical outcomes.

Recently, the gut microbiota has emerged as a key factor in RE pathogenesis. Ionizing radiation damages microbial composition and function, contributing to barrier dysfunction, inflammatory responses, immune imbalance, and altered metabolism [5,6]. Beyond pathophysiology, the microbiome offers novel opportunities for risk prediction, dynamic monitoring, biomarker discovery, and stratified interventions [7–9]. Compared with traditional clinical factors and dosimetric indicators, microbiome profiles may offer additional biological information for identifying high-risk populations, revealing potential clues regarding the transition from acute injury to chronic persistence, and aiding in the development of more personalized microbiome intervention strategies. However, current research faces challenges that include heterogeneous endpoints, limited reproducibility, and sparse clinical translation. Although recent reviews have outlined microbial stratification and intervention for RE [8,9], the present review complements existing literature by providing a more focused evaluation of their translational implementation. Rather than solely relying on cross-sectional baseline biomarkers, we discuss the predictive potential of longitudinal multi-omics data and machine learning workflows while addressing key methodological limitations. Furthermore, we expand the therapeutic scope beyond standard probiotics and fecal microbiota transplantation (FMT) to examine emerging strategies, including engineered biotherapeutics and advanced delivery systems, and discuss how integrating multi-disciplinary tools could help optimize personalized microbiome-guided management (Fig. 1).

## Methods

### Literature search strategy

This article is formulated as a comprehensive narrative review. A literature search was conducted for articles published between January 2006 and May 2026 using the PubMed database. To capture relevant literature regarding both predictive biomarkers and therapeutic advancements in RE, keywords related to “radiation enteritis”, “radiation-induced intestinal injury”, and “radiation proctitis” were paired with terms encompassing microbial profiling (“gut microbiota”, “dysbiosis”, “multi-omics”, and “machine learning”) and interventions (“probiotics”, “fecal microbiota transplantation”, and “engineered bacteria”) (Table 1). The retrieved literature was sequentially screened for clinical and mechanistic relevance, and the reference lists of pivotal trials and reviews were cross-checked via snowball searching.

**Table 1.** PubMed search string used in literature search

Study focus	Search string
Microbial mechanisms, pathogenesis, and risk stratification in RE	(((Radiation Enteritis[MeSH Terms]) OR (“radiation enteritis”[Title/Abstract]) OR (“radiation-induced intestinal injury”[Title/Abstract]) OR (“radiation enteropathy”[Title/Abstract]) OR (“radiation proctitis”[Title/Abstract]))) AND (((Gastrointestinal Microbiome[MeSH Terms]) OR (“gut microbiota”[Title/Abstract]) OR (“gut microbiome”[Title/Abstract]) OR (“dysbiosis”[Title/Abstract]) OR (“biomarker”[Title/Abstract]) OR (“risk stratification”[Title/Abstract]) OR (“prediction”[Title/Abstract]) OR (“metagenomics”[Title/Abstract]) OR (“multi-omics”[Title/Abstract])))
Evidence regarding microbiome-targeted biotherapeutics and interventions	(((Radiation Enteritis[MeSH Terms]) OR (“radiation enteritis”[Title/Abstract]) OR (“radiation-induced intestinal injury”[Title/Abstract]) OR (“radiation proctitis”[Title/Abstract]))) AND (((Probiotics[MeSH Terms]) OR (“probiotics”[Title/Abstract]) OR (Fecal Microbiota Transplantation[MeSH Terms]) OR (“fecal microbiota transplantation”[Title/Abstract]) OR (“FMT”[Title/Abstract]) OR (“engineered bacteria”[Title/Abstract]) OR (“live biotherapeutic products”[Title/Abstract]) OR (“synbiotics”[Title/Abstract]) OR (“microbial consortium”[Title/Abstract])))

### Inclusion and exclusion criteria

Specific eligibility criteria were established to guide literature selection, ensuring clinical relevance and mechanistic depth. Studies were included if they met the following benchmarks: (a) original research articles (including prospective or retrospective clinical cohorts, case–control studies, and established animal models) investigating the role the gut microbiota plays in RE; (b) studies characterizing baseline or dynamic temporal alterations in microbial diversity, taxonomic composition, and functional metabolic profiles [e.g., short-chain fatty acids (SCFAs), tryptophan derivatives, and bile acids] under radiation exposure; (c) studies developing or validating machine learning, radiomics, or multi-omics integrated predictive frameworks for toxicity stratification; or (d) studies evaluating the preventative or therapeutic efficacy of microbiome-targeted interventions, including traditional probiotics/prebiotics, FMT, and next-generation engineered biotherapeutics.

Conversely, publications were excluded if they met any of the following criteria: (a) studies investigating chemotherapy- or immunotherapy-induced mucositis without a concurrent radiotherapy regimen; (b) studies lacking explicit data regarding microbial alterations or concrete clinical/pathological endpoints; and (c) non-English publications.

### Data extraction and literature review

Data on microbial alterations and pathogenesis were reviewed and summarized, focusing on biological sample types, sequencing platforms, and radiation-induced dynamic shifts in microbial diversity and taxonomic composition. Baseline microbial signatures, key metabolic biomarkers, and the performance metrics of integrated multi-omics or machine learning predictive frameworks were compiled for literature addressing risk stratification. Furthermore, detailed characteristics of microbiome-modulating therapies were extracted, categorizing interventions into traditional probiotics, FMT, and next-generation engineered biotherapeutics, such as probiotic–drug conjugates. For these interventional studies, the extraction outcomes specifically focused on targeted delivery efficiency, mucosal barrier repair, and overall radioprotective efficacy.

## Gut Microbiota and the Pathophysiological Mechanisms of RE

### Host-damage basis of RE

Although radiotherapy kills tumor cells, it inevitably damages the highly radiation-sensitive intestinal epithelial tissue. This process is no longer viewed as mere physical destruction but rather as a dynamic response network involving the “microbiota–epithelium–immune” axis. Ionizing radiation causes intestinal epithelial cell apoptosis by directly inducing DNA damage, indirectly generating large amounts of reactive oxygen species (ROS) [10,11], destroying crypt structures. Studies have shown that radiation markedly down-regulates the Wnt/β-catenin signaling pathway and inhibits the proliferation and differentiation of Lgr5^+^ stem cells, leading to crypt atrophy and the loss of intestinal mucosal repair mechanisms [12–14]. Concurrently, a reduction in goblet cells and decreased MUC2 expression further increase intestinal permeability, allowing microorganisms and their components within the intestinal lumen to come into closer contact with the mucosa and lamina propria, thereby increasing the risk of infection [15–17], and triggering the release of damage-associated molecular patterns (DAMPs) from damaged cells.

### Radiation-driven ecological reprogramming and functional loss of the gut microbiota

Following the aforementioned barrier disruption, the effects of ionizing radiation on the gut microbiota constitute a process of ecological reprogramming that is radiation-specific and dose- and time-dependent. Radiation can directly penetrate microbial cells, causing DNA damage; however, considerable differences in radiation sensitivity occur among different microbial communities [10,11].

Radiation-sensitive members of the Firmicutes phylum, particularly butyrate-producing genera such as *Faecalibacterium prausnitzii* and *Roseburia*, exhibit a marked decline in abundance following radiation therapy. These microbial communities serve as the primary energy source for intestinal epithelial cells, and their reduction directly leads to impaired intestinal barrier repair capacity and weakened anti-inflammatory effects [18]. Conversely, certain radiation-tolerant facultative anaerobes, such as those of the Proteobacteria phylum and its subgenera, *Escherichia and Shigella*, as well as Enterococcus, may increase in relative abundance following reduced competitive pressure [19].

**Fig. 2.**
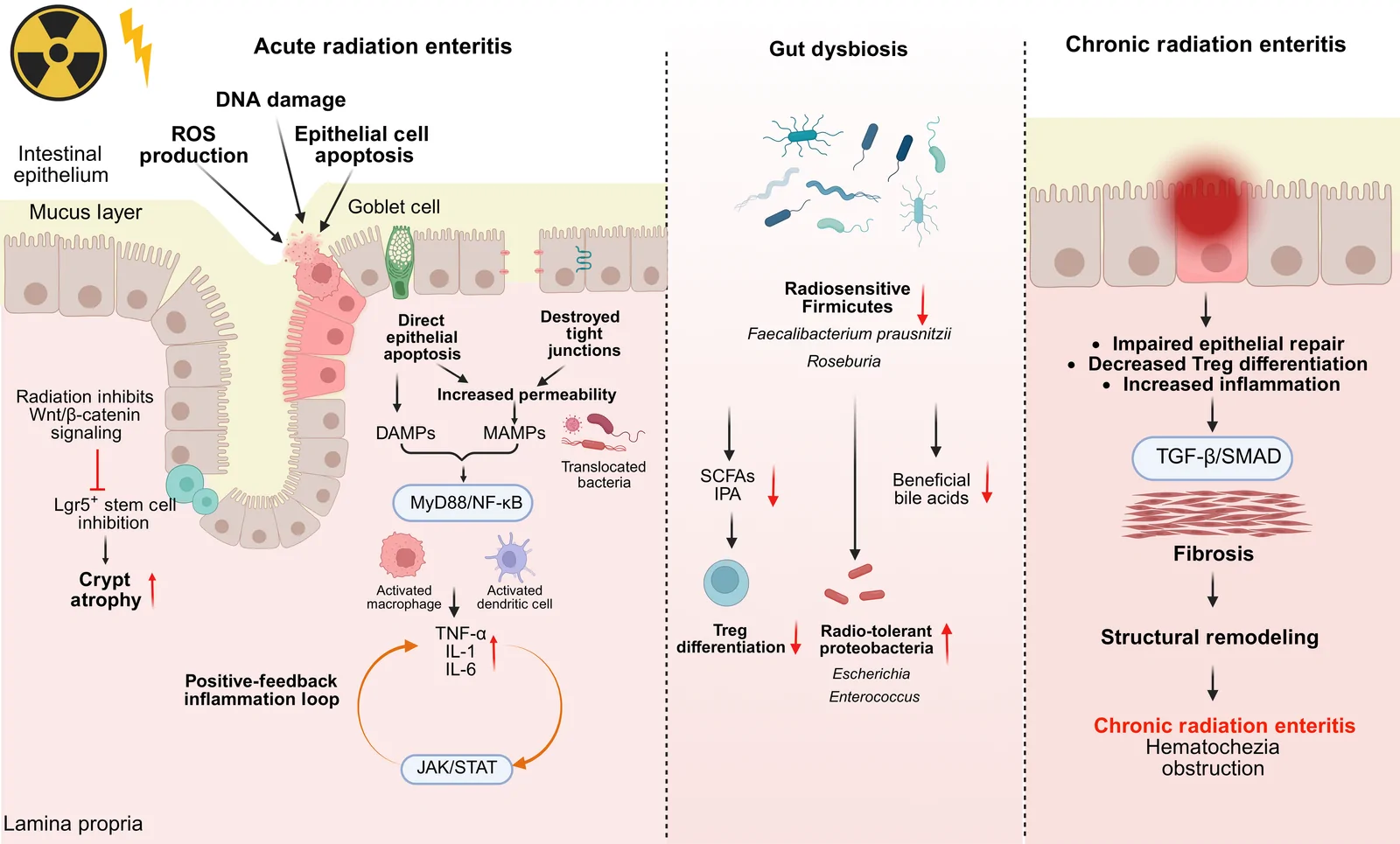
The cascading mechanism of RE: Interplay between epithelial barrier destruction, microbial dysbiosis, and immune-mediated fibrosis. (Left) Epithelial barrier destruction: Radiation induces epithelial cell apoptosis and crypt stem cell damage, destroying tight junctions and promoting bacterial translocation. This triggers the self-amplification inflammatory cycle mediated by the MyD88 and JAK/STAT signaling pathways. (Middle) Microbials and metabolic dysbiosis: Radiation therapy reduces radiosensitive bacteria while increasing the number of opportunistic proteobacteria, resulting in a substantial loss of protective microbial metabolites. (Right) Chronic remodeling and fibrosis: Continuous metabolic depletion and inflammatory signals inhibit Treg differentiation and impair epithelial repair. Chronic activation of the TGF-β/SMAD pathway ultimately drives tissue fibrosis and structural remodeling.

Temporal remodeling of the microbial community is accompanied by substantial functional changes, including reduced SCFAs, diminished immunomodulatory capacity, and delayed mucosal repair [20]. Microbiota-derived metabolites, particularly SCFAs, indole-3-propionic acid (IPA), and secondary bile acids, play crucial roles in maintaining the epithelial barrier, regulating local immunity, and promoting tissue repair. Depletion of these anti-inflammatory metabolites, combined with compromised barrier integrity and the overgrowth of pathogenic bacteria, allows translocated bacteria and microbiota-associated molecular patterns (MAMPs) to enter the lamina propria. Together with host DAMPs, they robustly activate nuclear factor-κB (NF-κB) and mitogen-activated protein kinase (MAPK) via MyD88-dependent signaling pathways, inducing the production of pro-inflammatory factors such as tumor necrosis factor-α (TNF-α), interleukin-1β (IL-1β), and IL-6. Furthermore, these factors activate the Janus kinase (JAK)/signal transducer and activator of transcription (STAT) pathway, forming a self-amplifying inflammatory loop that exacerbates the local inflammatory response [21–23]. If this bacteria-driven inflammation persists, it can activate the transforming growth factor-β (TGF-β)/SMAD pathway, among others, leading to fibrosis and chronic structural remodeling, which trigger major symptoms such as intestinal dysfunction, bloody stools, and intestinal obstruction [24] (Fig. 2).

## Pre-Radiotherapy Gut Microbiota as a Tool for Predicting the Risk of RE

### Baseline microbiota characteristics and susceptibility to RE

Individual variations in pre-radiotherapy gut microbiota strongly influence radiation susceptibility. With the advancement of high-throughput sequencing, the baseline microbial diversity, specific taxa abundance, and functional metabolic profiles have emerged as promising indicators for early RE risk prediction and clinical stratification [8,25,26]. To consolidate the current clinical evidence, Table 2 summarizes recent human observational and prospective cohorts investigating microbiome predictors and baseline signatures for RE.

**Table 2.** Summary of human studies evaluating gut microbiome predictors for RE

Study design	Malignancy type/radiation site	Sample size (*N*)	Analytical techniques	Key baseline predictive signatures	Major conclusions related to RE	Reference
Prospective observational multi-cohort	Prostate and pelvic cancers/pelvic region	134	16S rRNA sequencing and metataxonomics	Decreased pre-RT microbial diversity; enrichment of specific SCFA producers (*Clostridium IV*, *Roseburia*, *Phascolarctobacterium*).	Alterations in the gut microbiota are strongly associated with both acute and late radiation enteropathy, providing a window for pretreatment risk stratification.	[29]
Prospective observational cohort	Cervical cancer/pelvic region	18	16S rRNA sequencing	Elevated baseline abundance of the genus Coprococcus; markedly reduced α-diversity post-RT	Intestinal dysbiosis actively contributes to the development and progression of RE. Pre-radiotherapy *Coprococcus* levels hold potential as a predictive biomarker.	[32]
Prospective observational cohort	Prostate cancer/pelvic/prostate region	215 (136 discovery, 79 validation)	16S rRNA sequencing	Decreased baseline α-diversity; altered core genera profile (*Faecalibacterium*, *Bacteroides*, *Parabacteroides*, *Alistipes*, *Prevotella*, *Phascolarctobacterium*)	Baseline microbiota signatures effectively predict acute GI toxicity risk. Machine learning decision trees successfully stratify patients into high- and low-risk categories.	[26]
Prospective observational cohort	Cervical cancer/pelvic region	65 (50 patients, 15 healthy controls)	16S rRNA sequencing	Pre-radiotherapy dominance of *Faecalibacterium* enterotype (Enterotype-3) and *Escherichia-Shigella* enterotype (Enterotype-2).	Specific gut enterotypes, rather than single bacterial genera or the traditional F/B ratio, serve as superior predictors for the occurrence of SARE.	[87]
Prospective observational cohort	Locally advanced cervical cancer/pelvic region	83	16S rRNA sequencing	Enrichment of Lachnospiraceae, Blautia, and Dorea; reduced Firmicutes/Bacteroidetes ratio and decreased α-diversity	Severe radiation-induced diarrhea strongly correlates with baseline intestinal dysbiosis, reduced microbial diversity, and the enrichment of pro-inflammatory taxa.	[88]
Prospective observational cohort	Cervical cancer/pelvic region	50	16S rRNA sequencing and LC-MS metabolomics	Baseline enterotypes dominated by *Faecalibacterium* (Enterotype-3) or *Escherichia-Shigella* (Enterotype-2); Fecal phenylethylamine levels	Integration of microbial enterotypes, metabolic signatures (phenylethylamine), and clinical features yields a highly accurate nomogram for predicting SARE.	[53]
Cross-sectional observational feasibility study	Pelvic malignancies/pelvic region	16	16S rRNA sequencing and GC-MS metabolomics	Lower baseline α-diversity; reduced baseline fecal volatile organic compounds (specifically heptanal and octanal)	Diminished baseline α-diversity possesses strong predictive value for GI toxicity. Fecal metabolomics offer complementary predictive efficacy alongside microbiome sequencing.	[89]

GC-MS, gas chromatography–mass spectrometry; GI, gastrointestinal; LC-MS, liquid chromatography–mass spectrometry; RE, radiation enteritis; RT, radiotherapy; SARE, severe acute radiation enteritis

#### Microbial diversity

Microbial diversity is a key indicator of gut microbiome homeostasis. Generally, a more diverse microbial community translates to enhanced stability and stronger mucosal barrier defenses [27]. In contrast, dysbiosis frequently presents as a drop in overall diversity, along with a shift toward pathogenic populations and loss of beneficial commensals. This imbalance inherently compromises the epithelial barrier and increases pro-inflammatory cytokine levels [28]. In patient cohorts, pretreatment with low microbial diversity is consistently associated with an increased susceptibility to RE [29]. Rather than acting as a direct mechanistic driver, this reduced baseline diversity primarily serves as a predictive biomarker, reflecting a “low homeostatic reserve” and frequently acting as a downstream consequence of prior physiological stress, thus predisposing the gut to barrier fragility.

Strong mechanistic foundations support this predictive capacity. A diverse microbiota helps maintain SCFA production, promotes regulatory T (Treg) cell differentiation, suppresses excessive colonization by pathogenic bacteria, and sustains intestinal mucosal barrier function [20,30]. When microbial diversity is insufficient, the gut is more prone to barrier fragility, increased endotoxin exposure, and activation of pro-inflammatory signaling pathways [31]. Consequently, patients with reduced baseline diversity may possess compromised gastrointestinal defenses, heightening their vulnerability to acute radiation injury.

Although microbial diversity has the advantages of being holistic and easily quantifiable, its clinical specificity is limited and easily affected by factors such as diet, antibiotic use, tumor type, and sequencing methods. Therefore, diversity indicators are more suitable as the basic signals for risk assessment than they are as independent clinical prediction tool.

#### Characteristics of specific microbial communities

The characteristics of specific gut microbiota are closely related to the severity of damage caused by radiation, and changes in the abundance of some key microbial communities before radiotherapy may indicate different risks of RE. Overall, a decrease in the number of microbial communities that provide barrier protection, anti-inflammatory effects, and metabolic support often indicates higher susceptibility; conversely, the enrichment of inflammatory-related microbial communities or opportunistic pathogens may be associated with a higher risk of toxicity.

##### Decreased beneficial bacteria and increased risk

The onset of RE is often accompanied by a reduction in the abundance of key probiotic bacteria, causing the defense system of the intestinal barrier fail. *Lactobacillus* and *Bifidobacterium*, 2 traditional probiotic genera, play central roles in maintaining intestinal barrier integrity and regulating local immunity [32]. These bacterial populations can induce intestinal epithelial cells to secrete mucins and tight junction proteins (such as occludin and ZO-1), thereby repairing the barrier damage caused by radiotherapy [32]. Studies have shown that their relatively low abundance in the gut before radiotherapy may indicate a higher risk of RE [33–35]. Similarly, butyrate-producing bacteria such as *F. prausnitzii* have also attracted widespread attention. Butyrate not only serves as a vital energy source for intestinal epithelial cells but also reduces pro-inflammatory cytokine expression by inhibiting inflammatory pathways such as the NF-κB pathway [7,32]; therefore, its depletion may indicate a decline in the anti-inflammatory and reparative capacity of the gut. Overall, the reduction in beneficial anaerobes and butyrate-producing bacteria before radiotherapy may suggest that patients lack sufficient microbiome protective reserves.

##### Potential value and controversy surrounding *Akkermansia muciniphila* (preclinical evidence)

*Akkermansia muciniphila* is an anaerobic gram-negative bacterium that resides in the intestinal mucus layer. It plays a crucial role in maintaining intestinal barrier function and regulating microbial homeostasis. By degrading mucins, *A. muciniphila* helps maintain the thickness of the intestinal mucus layer and enhances intestinal barrier function [36]. Some studies have suggested that a decrease in *A. muciniphila* abundance before radiotherapy may be associated with the onset and progression of RE [7,8]. He *et al*. [15] reported that in a mouse model, this bacterium can alleviate RE by increasing propionate levels and up-regulating the expression of tight junction proteins and MUC2. Qu *et al*. [37] found that *A. muciniphila* can improve colitis in Toll-like receptor 4 (TLR4)-deficient mice by increasing the proportion of RORγt^+^ Treg cells and activating their mediated immune response.

However, current research on the role of *A. muciniphila* is not consistent. A recent study by Wang *et al*. [16] suggests that *A. muciniphila* may exert a “double-edged sword” effect in RE: In the microenvironment of radiation-induced intestinal injury, an increase in the abundance of *A. muciniphila* may instead create a vicious cycle by depleting mucin, enriching pathogenic bacteria, and inducing pro-inflammatory responses, thereby exacerbating intestinal injury; conversely, FMT can alleviate RE symptoms by reducing their abundance of *A. muciniphila*. Importantly, these controversial findings, including the “double-edged sword” effect, are currently derived exclusively from *in vivo* murine models. Their underlying mechanistic divergence likely hinges on several context-dependent variables. Under radiation-induced mucin starvation, in which host goblet cell function is severely impaired, the constitutive mucolytic activity of *A. muciniphila* can surpass the critical mucus depletion threshold, transitioning from homeostatic turnover to direct barrier destruction [38]. Furthermore, the resultant release of mucus oligosaccharides can inadvertently foster niche competition and cross-feeding, thereby enriching opportunistic pathogens [39,40]. This metabolic shift, compounded by an altered tissue milieu that diminishes protective SCFA utilization, is further modulated by the baseline host immune state, which ultimately dictates whether the bacterium prompts regulatory tolerance or amplifies the prevailing inflammatory circuitry. Consequently, the translational utility of *A. muciniphila* as a predictive biomarker requires meticulous context-specific interpretation and cannot be simplified as a unidirectional protective indicator without rigorous validation in human clinical cohorts [41].

##### Enrichment of pathogenic bacteria and high-risk signals

Observational clinical studies on patients with RE have shown that the abundance of opportunistic bacteria is often abnormally elevated, serving as a biomarker of disease progression. Specifically, the enrichment of *Proteobacteria* and its gram-negative taxa is a prominent correlative signature [32]. While *in vitro* and animal models suggest that their lipopolysaccharides can actively promote inflammation via the TLR4/MyD88/NF-κB pathway [42,43], current human data position their baseline elevation primarily as a downstream consequence of altered gut oxygenation or barrier fragility, rather than as a primary driver. Furthermore, the abnormal proliferation of certain opportunistic pathogens, such as *Clostridioides difficile*, may increase the risk of developing or exacerbating RE, particularly in patients with a history of antibiotic exposure or preexisting disruption of intestinal homeostasis [44–46]. This risk suggests that changes in the abundance of potentially pathogenic bacteria before radiotherapy may not only reflect the high-risk microbiome status of a patient but also provide early warning information for subsequent toxicity management.

However, most current evidence have been obtained from small sample studies or animal experiments, and specific microbial signals remain inconsistent across studies. Therefore, the baseline microbiota is more suitable as a candidate biological layer for predicting RE risk, whereas a future direction may involve integrating microbial characteristics with dosimetric parameters, clinical features, and metabolite markers to explore predictive models with improved reproducibility.

### Dynamic changes in the gut microbiome during radiotherapy and the predictive value of functional biomarkers

During radiotherapy, the structure and function of the gut microbiome undergo profound remodeling over time. These dynamic changes are closely associated with the risk of acute RE, offering new possibilities for early toxicity warnings based on microbiome pathways.

Diversity indices commonly show a sustained downward trend during radiotherapy, accompanied by a marked shift in gut microbiota composition [6,47,48]. The relative abundance of probiotic genera, such as *Lactobacillus* and *Bifidobacterium*, generally declines as radiotherapy progresses [34,35], whereas that of the Proteobacteria phylum and opportunistic pathogens, such as *Escherichia* and *Shigella*, markedly increases. Wang *et al*. [32] found that in patients with cervical cancer undergoing radiotherapy, the microbiota of those of RE was characterized by a markedly reduced α-diversity and increased abundance of *Proteobacteria*; furthermore, microbiota derived from patients with RE could induce epithelial inflammation and barrier dysfunction *in vitro*, enhancing the expression of TNF-α and IL-1β. Notably microbiota remodeling did not follow a unidirectional pattern in any of the radiotherapy scenarios. Chen *et al*. [49] observed a unique “first increase, then decrease” pattern in observational patient cohorts undergoing radiotherapy, in which diversity actually increased during the middle phase of treatment, and most microbiota returned to baseline levels after treatment completion. This finding suggests that gut microbiota distant from the irradiation field may possess a capacity for homeostatic regulation, with its response pattern constrained by the spatial relationship between the irradiated target area and intestine.

Temporal remodeling of the microbial structure is accompanied by changes in functional metabolic output. SCFAs are core metabolites of the intestinal anaerobic microbiota that maintain intestinal barrier homeostasis by promoting tight junction protein expression, enhancing epithelial cell energy metabolism, and inducing Treg differentiation [20]. Lu *et al*. [50] demonstrated in a mouse model that a marked reduction in butyrate-producing bacteria (*Ruminococcaceae*, *Lachnospiraceae_NK4A136*, etc.) occurred during the acute injury phase, accompanied by elevated fecal lipopolysaccharide levels and activation of the TLR4/MyD88/NF-κB inflammatory signaling cascade. Concurrently, KEGG pathway enrichment analysis revealed that multiple pathways, including tryptophan metabolism and primary bile acid synthesis, were substantially affected. Li *et al*. [51] further revealed substantial disruptions in glycerophospholipid metabolism during the active phase of RE through a combined microbiome-lipidomics analysis and identified 5 pairs of functional associations between bacteria and metabolites. The resulting decreased metabolite levels are initially a downstream consequence of microbial ecological collapse; however, this depletion subsequently acts as a functional biomarker that often precedes the onset of clinical symptoms.

These dynamic and functional dual-level microbiome changes have a potential predictive value. Compared with a single baseline microbiome composition, time-series microbiome trajectories and functional and metabolomic changes often provide earlier and more sensitive indications of acute toxicity risk, particularly when no obvious clinical manifestations appeared within a few weeks of radiotherapy. From a clinical translation perspective, incorporating dynamic microbiome indicators into predictive models has the potential to complement traditional risk factors; however, the ability to enhance the timeliness and accuracy of RE risk assessments requires validation in larger cohorts [8,52].

### Development, validation, and challenges of predictive models

Based on the aforementioned patterns of dynamic gut microbiome remodeling during radiotherapy, the key to advancing clinical translation in this field lies in integrating multidimensional microbiome information into clinically actionable toxicity prediction tools. Although current studies mainly rely on 16S rRNA gene sequencing to profile microbiota composition, its low taxonomic resolution limits the accuracy of functional mapping. The multi-omics integration strategy combines metagenomics, metatranscriptomics, and metabolomics, and incorporates host genetics, clinical characteristics, and detailed dosimetric parameters into a unified framework, which is crucial for maximizing the predictive accuracy of the model.

#### Comparison of existing prediction models

Recent efforts have combined multimodal datasets to construct machine learning algorithms predicting RE risk (Table 3). However, the existing studies were found to be highly heterogeneous in cohort size, algorithm selection, and validation rigor. For example, Iacovacci *et al*.’s research model [26] can effectively predict the risk of gastrointestinal toxicity after radiotherapy in both internal and external validation cohorts, while other studies only rely on internal cross-validation or calibration curves, which seriously limits their generalizability [53–55].

**Table 3.** Summary of machine learning predictive models for RE risk

Cancer type and therapy	Cohort size	Sampling time point	Data type	Outcome definition	Algorithm and validation strategy	Performance metrics	Clinical limitations	Reference
Prostate cancer (RT)	*n* = 247 (136 discovery, 79 validation, 32 external)	Pre-radiotherapy (baseline)	16S rRNA	Persistent acute GI toxicity (average CTCAE grade > 1.3)	Decision tree; independent external validation	Identified high-risk class with 60% (internal) and 33% (external) toxicity rates	Taxonomic resolution limited to genus level; geographic bias limits generalization.	[26]
Cervical cancer (VMAT)	*n* = 50	Dynamic (pre-RT and post-RT at weeks 2, 4, 6)	16S rRNA, LC-MS, ELISA, CBA	Severe acute RE (combined score ≥ 3)	Logistic regression nomogram; internal calibration and DCA	AUC: 0.975 (95% CI: 0.953–0.998)	Small sample size; lacks independent multi-center external validation.	[53]
Cervical cancer (RT)	*n* = 386 (270 training, 116 test)	Pre-radiotherapy (baseline)	Clinical, laboratory, and dosimetric data	Acute RE (RTOG criteria during or ≤3 months post-RT)	Random forest (best), DT, LR; internal split-sample validation	RF model AUC: 0.961, sensitivity: 0.934	Single-center; excluded patients with severe comorbidities; poor “black box” interpretability.	[54]
Cervical cancer (Post-op RT)	*n* = 178 (124 training, 54 validation)	Pre-radiotherapy (baseline)	CT radiomics, clinical, dose-volume data	Severe radiation proctitis (RTOG/EORTC ≥ grade 2)	Logistic regression with SHAP; internal split-sample validation	Radiomic model AUC: 0.6855	Indirect inference of microbiota; retrospective design; single-center.	[55]

VMAT, volumetric modulated arc therapy; CTCAE, Common Terminology Criteria for Adverse Events; RTOG, Radiation Therapy Oncology Group; EORTC, European Organization for Research and Treatment of Cancer; LC-MS, liquid chromatography–mass spectrometry; CBA, cytometric bead array; DT, decision tree; LR, logistic regression; RF, random forest; DCA, decision curve analysis; SHAP, SHapley Additive exPlanations

Although these models have shown some predictive power, translating them into clinical applications remains challenging. Microbiome datasets are compositional (representing relative rather than absolute abundances), zero-inflated, and uniquely susceptible to batch effects and feature instability across sequencing runs. To build reliable models, researchers must employ rigorous compositional data analysis (CoDA) methods and robust normalization techniques before model training [56,57]. In addition, the superposition of small sample size with high-dimensional feature space exacerbates the overfitting risk. In this process, strict methodological specifications should be maintained to prevent data leakage in cross-validation, and advanced sampling techniques should be used to deal with class imbalance, such as the small proportion of severe toxicity cases. Most crucially, predictive performance in a discovery cohort is insufficient, and strict, independent external validation is mandatory to prove clinical utility and generalizability.

#### Advancing from correlative prediction to biological causal validation

The essential distinction between predictive performance and biological interpretability must be clearly articulated in research. Although some existing machine learning models can achieve certain prediction accuracy, these algorithms fundamentally reveal statistical correlations rather than biological causality [58]. Models may identify a particular bacterial taxon as a highly weighted feature predicting RE risk, but this does not distinguish whether the organism is a driver driving disease development or a passenger simply responding to an altered gut microenvironment.

To definitively elucidate causal mechanisms, artificial intelligence (AI) predictions must be integrated with fundamental biological approaches. For example, using *in vitro* organoid–microbiota coculture technology, researchers can directly apply core strains or metabolites selected by AI to human intestinal epithelial models to experimentally confirm their roles in mucosal injury or repair [59]. Moreover, the introduction of causal inference methods into computational models will further help isolate true biological drivers from confounding variables.

#### Explainable AI and longitudinal dynamics

Although machine learning models can provide some predictive reference, their “black box” nature makes it difficult to explain the underlying biological mechanism, which affects clinicians’ trust and willingness to adopt them. Explainable AI (XAI) frameworks, in particular SHAP (SHapley Additive exPlanations) [60] and LIME (Local interpretable Model-agnostic Explanations), are expected to effectively advance the solution to this problem by providing interpretability at the feature level. The core advantage of these frameworks lies in the stratified resolution of both cohort-wide patterns and individual characteristics. At the macro level, they can efficiently screen out generalizable microbial biomarkers across the entire cohort. At the micro level, the complex interaction between baseline gut microbiota and dosimetric parameters of a specific patient can be deeply dissected so as to accurately elucidate the mechanism of generating individualized risk scores [61,62]. However, applying XAI to microbiome data requires careful handling of multicollinearity and the mathematical dependencies introduced by log-ratio transformations, an area necessitating specialized *post hoc* explanation techniques.

Furthermore, most of the existing models are highly dependent on cross-sectional baseline data ignoring the dynamic temporal information captured through longitudinal sampling. Future clinical applications must employ more advanced computational architectures, such as longitudinal mixed-effects modeling and graph-based microbial interaction networks, to track the evolution of microbiota during RT. Accurate identification of these dynamic and modifiable microbial tipping points and optimal therapeutic windows will ultimately promote a shift in the clinical paradigm from static and passive toxicity prediction to active and adaptive preventive intervention.

## Microbiome-Driven Personalized Radiotherapy Tolerance and Intervention Strategies

In recent years, microbiome-based therapies such as probiotics, prebiotics, and FMT have emerged as promising strategies to mitigate the adverse effects of radiotherapy and enhance its efficacy [63]. These therapies aim to modulate the gut microbiota, which plays a critical role in maintaining immune homeostasis, tissue repair, and inflammation regulation, all of which are compromised during radiotherapy.

Although diverse microbiome-based therapies are being investigated, recognizing the marked differences in their clinical maturity is important. Currently, these interventions can be broadly divided into 2 categories: approaches that have entered clinical trials or routine practice (such as traditional probiotics, prebiotics, and FMT) and experimental preclinical platforms (such as coated probiotics and live engineered biotherapeutics). Furthermore, applying these live therapies to oncology patients requires careful consideration of safety issues, as these individuals are frequently immunocompromised or receiving combined chemoradiotherapy (Table 4).

**Table 4.** Summary of microbiome-targeted interventions for RE categorized by level of evidence

Evidence level	Intervention type	Key findings	Reference
Randomized controlled trials (RCTs)	Probiotics: *L. plantarum*	Reduced the number of days with loose stools and alleviated abdominal pain and defecation urgency in pelvic RT	[65]
	Prebiotics: Inulin and fructo-oligosaccharide	Improved stool consistency and reduced days of watery stools during abdominal RT	[67]
	Probiotics: *L. acidophilus* and *Bifidobacterium*	Reduced the incidence and severity of abdominal pain and diarrhea in cervical cancer patients undergoing RT	[64]
Observational human studies	FMT Variant: Washed microbiota transplantation	Prospective cohort study showed markedly higher clinical response rates and mucosal healing in RE patients compared to routine therapies	[90]
Case reports	FMT: Traditional fecal microbiota transplantation	Effectively alleviated clinical symptoms of chronic RE and improved long-term quality of life	[75]
Animal studies (*in vivo*)	Engineered probiotics: LR-IL-22	Mitigated radiation damage, protected microvascular endothelial cells and lamina propria, and improved survival following whole abdomen irradiation	[68]
	Engineered probiotics: Hb-auWMT (*H. biformis*-augmented WMT)	Mitigated radiation-induced injury, increased Treg cells, and elevated protective SCFAs	[90]
	Modified probiotics: LGG@CT@CA	Promoted long-term colonic retention, scavenged ROS, and successfully alleviated radiation-induced small intestinal damage	[69]
	Microcarrier system: SP@AMF	Delivered radioprotectant to the whole small intestine, preventing early/delayed injury and improving survival without compromising tumor radiotherapy	[70]
	Metabolite replenishment: FMT and IPA	Enhanced intestinal barrier function, suppressed inflammatory responses, and reduced ROS levels	[74]
*In vitro* studies	Microcarrier system: SP@AMF on IEC-6 cells	Demonstrated lower toxicity and more effective protection against DNA double-strand breaks in irradiated normal epithelial cells	[70]
	Microfluidics: Human gut-on-a-chip	Modeled radiation injury-induced cell death and evaluated countermeasure drug responses in a highly controlled environment	[10]

### Probiotics and prebiotics

Multiple clinical studies have demonstrated the preventive and therapeutic effects of probiotics, prebiotics, and synbiotics on RE [8]. A clinical trial conducted by Linn *et al*. [64] demonstrated that patients undergoing radiotherapy supplemented with *Lactobacillus acidophilus* and *Bifidobacterium* experienced a marked reduction in the incidence of abdominal pain and diarrhea. This finding suggests that probiotic supplements may serve as effective means of reducing the frequency and severity of radiotherapy-associated diarrhea in patients with cervical cancer. Another trial conducted by Ahrén *et al*. [65] showed that the intake of *Lactobacillus plantarum 299* and *Lactobacillus plantarum HEAL9* alleviated gastrointestinal symptoms and reduced radiation toxicity in women with gynecological cancers undergoing pelvic radiotherapy. A systematic review and meta-analysis of randomized controlled trials showed that probiotics can reduce the risk of radiation-induced diarrhea by approximately 40% [66]. Garcia-Peris *et al*. [67] conducted a randomized, double-blind, placebo-controlled trial involving 38 patients with gynecological cancer receiving abdominal radiotherapy. During radiotherapy, both groups experienced an increase in monthly bowel movements; however, the prebiotic group had fewer days of watery stools than the placebo group, indicating that prebiotics can improve stool consistency in patients undergoing radiotherapy.

Additionally, drugs can be used in combination with probiotics to enhance their therapeutic effects. For example, the second-generation probiotic *Lactobacillus reuteri-IL-22*, developed using *L. reuteri* as a platform, enhances the delivery of IL-22 to the irradiated intestine, thereby showing the potential to alleviate radiation-induced intestinal damage in preclinical models [68]. Sun *et al*. [69] reported a novel strategy integrating a bacterial bilayer coating with hydrogel microsphere encapsulation technology to improve the colonic retention and colonization efficiency of *Lactobacillus rhamnosus*, which can mitigate radiation-induced small intestinal damage and improve small intestinal mucosal survival rates in animal models. Furthermore, an oral delivery system constructed using the microalga Spirulina (an edible microorganism) as a carrier can deliver the selective normal tissue radioprotectant amifostine to the small intestine, demonstrating drug accumulation and radiation protection in murine models. This method markedly outperformed free-form drugs and enteric-coated capsules in preventing radiation-induced intestinal damage and prolonging survival, without affecting tumor regression [70]. Despite these positive results, clinical research on probiotics remains limited. Most studies on the efficacy of specific probiotic strains in patients undergoing radiotherapy lack conclusive evidence or reliable data to support their conclusions. This limitation has created an important knowledge gap in the field of intestinal microbiome research. More comprehensive research and accurate clinical evaluation programs are thus required to verify the practical value of probiotics and other microbiome-based therapies in improving the effects of radiotherapy.

### Fecal microbiota transplantation

FMT has shown promising therapeutic potential in various conditions, including RE [71,72]. FMT directly modulates changes in the composition of the host microbiome and promotes overall health by transferring donor feces into the gut of a recipient. This innovative strategy targets diseases associated with microbial dysbiosis by modulating the gut microbiota, thereby alleviating radiation-induced gastrointestinal toxicity, maintaining gut bacterial composition, and preserving the mRNA and long noncoding RNA expression profiles in irradiated hosts [73].

Animal studies have demonstrated the therapeutic potential of FMT: It improves survival rates in irradiated animals, increases the white blood cell counts in peripheral blood, maintains intestinal epithelial integrity, improves gastrointestinal function, and effectively alleviates symptoms of chronic RE caused by intestinal and mucosal damage. FMT can profoundly alter the composition of the gut microbiota and alleviate radiation therapy-related symptoms of diarrhea and constipation [72]. FMT effectively mitigates RE by replenishing IPA, a key microbial metabolite in the gut. In preclinical studies, this therapy has been shown to enhance intestinal barrier function and epithelial integrity, suppress radiation-induced inflammatory responses, reduce ROS levels, and promote angiogenesis without accelerating tumor growth [74]. Furthermore, multiple case reports have indicated that FMT can effectively alleviate the clinical symptoms of chronic RE, enhance the quality of life of patients, and improve prognosis by modulating the gut microbiota composition [75]. These preliminary reports suggest that FMT may help improve clinical symptoms and potentially support long-term recovery after radiotherapy, although larger controlled human trials are necessary to validate these findings.

Nevertheless, the widespread clinical application of FMT in RE remains limited owing to donor batch heterogeneity and severe safety concerns [76]. Safety issues require deep consideration, particularly in oncology patients who are often immunocompromised due to underlying malignancies or concurrent chemoradiotherapy. In patients with neutropenia or severe radiation-induced mucosal barrier disruption, the introduction of undefined live microbial communities via FMT increases the risk of pathogen translocation, opportunistic infections, and fatal bacteremia [77]. Therefore, rigorous donor screening and a strategic shift toward rationally designed and defined microbial consortia are imperative to maximize therapeutic efficacy while minimizing unpredictable infectious risks.

### Modified bacterial preparations for the treatment of RE (preclinical models)

To overcome the harsh gastrointestinal environment, recent preclinical rodent models have increasingly utilized advanced delivery systems and surface modifications. Protective strategies, such as alginate-based hydrogels, mucus-penetrating microspheres, and nanoparticle coatings, have been developed to shield engineered strains (e.g., *Escherichia coli Nissle 1917*) from gastric acid and enhance colon adherence [78–81]. Although such advanced delivery systems are currently primarily validated in IBD models, they provide promising translational templates for overcoming delivery barriers in the RE microenvironment. Furthermore, genetic engineering approaches have enabled probiotics to overexpress specific anti-inflammatory substances, such as ROS-scavenging enzymes, thereby directly targeting intestinal inflammation [82]. Although these triggerable, probiotic–drug conjugates show promising diagnostic and therapeutic effects in animal models, their application remains strictly experimental.

Notably, unlike traditional probiotics or FMT, these modified bacterial preparations and live engineered biotherapeutics are currently confined to experimental preclinical platforms. The translation of these techniques to clinical applications requires a cautious attitude toward their safety and technical hurdles. In immunocompromised patients undergoing concurrent chemoradiotherapy, the administration of genetically modified live organisms may introduce severe risks of systemic infection and hyper-inflammation [83]. In addition, the dynamic gut environment can be exposed to ecological risks such as horizontal gene transfer to commensal flora and uncertain bacterial colonization [84]. Addressing these ecological concerns requires fail-safe biocontainment strategies, such as genetic “kill switches”, to ensure absolute post-treatment clearance [85]. Crucially, the translational realism of this field is further constrained by the technical challenges of scaling production to Good Manufacturing Practice (GMP) standards and navigating complex regulatory approval pathways before these platforms can safely enter early-phase clinical trials.

## Discussion

Although profound insights have been gained on the interaction between the gut microbiota and RE, translating these basic findings into clinical practice remains complicated by numerous confounding variables. Key variables such as diet, concomitant medications (e.g., antibiotics and proton pump inhibitors), systemic treatments (chemotherapy and immunotherapy), and rigorous bowel preparation can drastically alter the baseline microbiota prior to mapping. In addition, host-specific factors, such as age, body mass index, race, baseline inflammatory status, tumor type, radiotherapy modality, and circadian rhythm effects, also introduce substantial biological heterogeneity (Table 5). Future large-scale prospective cohort studies must rigorously control for or systematically stratify these confounders to improve the translational relevance and predictive accuracy of machine learning frameworks.

**Table 5.** Major confounding variables in microbiome studies of RE

Category	Confounding variables	Potential microbiome and host impact	Implications for RE modeling	Mitigation strategies	Reference
Medication	Antibiotics, proton pump inhibitors (PPIs)	Decrease microbial diversity; induce ectopic colonization of oral taxa in the gut	Introduce false-positive risk signals independent of radiation injury	Exclude patients with recent exposure, or incorporate as covariates in multivariate models	[91,92]
	Bowel preparation (laxatives/enemas)	Acutely deplete fecal microbial biomass; reshape mucosa-associated microbiota	Disrupt true baseline homeostasis, compromising predictive accuracy	Standardize sample collection strictly prior to any bowel cleansing procedures	[93]
Oncological	Chemotherapy, immunotherapy	Induce systemic inflammation; deplete commensal beneficial taxa	Overlapping treatment toxicities confound the isolation of radiation-specific effects	Stratify patient cohorts by treatment modality (e.g., RT alone *vs*. concurrent chemoradiotherapy)	[94]
	Tumor characteristics, radiation modalities	Cause heterogeneous anatomical alterations and localized tissue injury	Injury severity is heavily confounded by dose-volume histogram (DVH) parameters	Directly integrate dosimetric parameters into machine learning algorithms	[95,96]
Host-intrinsic	Age, BMI, ethnicity	Drive intrinsic heterogeneity in baseline microbiota composition	Lead to external validation failure of single-center models (poor generalizability)	Conduct multi-center studies; implement rigorous demographic matching for control groups	[97,98]
	Baseline inflammatory status	Pre-configure the host immune-microbiome axis	Lower the threshold for RE as an independent risk driver	Screen for baseline inflammatory biomarkers (e.g., CRP, fecal calprotectin)	[99]
Lifestyle	Dietary habits, circadian rhythms	Dictate baseline enterotypes; mediate cyclical oscillations in microbial abundance	Random sampling or nonstandardized diets introduce substantial statistical noise	Utilize dietary questionnaires; strictly standardize sample collection timing (e.g., early morning)	[100,101]

Furthermore, current research has focused almost exclusively on bacterial groups, and a notable lack of research on the composition of nonbacterial microbiomes remains. The gut ecosystem is highly complex, and fungi, viromes, and phages play key roles in regulating mucosal inflammation and host immune responses. For example, phages can directly shape bacterial community structures and drive temporal dysbiosis [86], whereas specific fungal species may synergistically exacerbate or mitigate radiation-induced barrier dysfunction. Investigating trans-kingdom microbial interactions represents a crucial direction for future research, as a holistic understanding of the entire microbial ecosystem is necessary to fully decode radiotherapeutic responses and develop comprehensive biological interventions.

Finally, several methodological constraints of this review must be explicitly acknowledged. Although we employed a structured literature search process, this review lacks a formal quality or risk-of-bias assessment of the included primary studies. Furthermore, many of the cited original studies are fundamentally limited by small sample sizes, single-center retrospective designs, or early preclinical models. Crucially, a profound “interspecies barrier” exists between rodents and humans. Preclinical models exhibit substantial inherent differences in baseline gut microbiota composition, radiosensitivity, metabolic rates, and immune system architecture compared with those of human patients. Consequently, successful microbial signatures or engineered biotherapeutics derived from rodent models do not automatically guarantee clinical efficacy in patients with cancer undergoing radiotherapy. Future research must cautiously contextualize these interspecies differences and temper translational claims until robust validation is achieved in well-designed, multicenter human clinical trials.

## Conclusion

In conclusion, the microbial ecosystem plays a crucial role in radiation tolerance and tissue damage, offering an innovative approach to predicting and mitigating the adverse effects associated with RE. However, transitioning these correlations into established clinical applications is currently accompanied by uncertainties. Present research remains heavily constrained by substantial methodological heterogeneity, small and single-center cohort sizes, and an array of clinical and host confounding factors that challenge the reproducibility and generalizability of microbial signatures. Furthermore, a formidable interspecies barrier separates preclinical rodent models from human patients, meaning that animal-derived mechanistic insights and therapeutic successes cannot be prematurely extrapolated to guarantee clinical efficacy. Therefore, although the translational potential of microbiome-targeted strategies is undeniable, their immediate clinical applicability must not be overstated. To safely bridge the gap between promise and reality, the field must systematically navigate these uncertainties through standardized multi-omics workflows, rigorous confounding controls, and large-scale, multi-center prospective human clinical trials.

## Data Availability

The data that support the findings of this study are included in the article or as supplementary materials.
